# Transcatheter edge-to-edge repair in anatomically complex degenerative mitral regurgitation: 3-year outcomes from a real-world registry

**DOI:** 10.1007/s00392-025-02644-1

**Published:** 2025-04-14

**Authors:** Nicoleta Nita, Michael Paukovitsch, Dominik Felbel, Matthias Gröger, Dominik Buckert, Mirjam Keßler, Wolfgang Rottbauer

**Affiliations:** Department of Internal Medicine II, University Medical Center, 89081 Ulm, Germany

**Keywords:** Complex mitral anatomy, Degenerative mitral regurgitation, M-TEER, Mitral prolapse, Commissure regurgitation, Mitral gradient

## Abstract

**Background:**

Recent developments in transcatheter mitral valve repair (M-TEER) have expanded the indication for the procedure beyond conventional criteria to include patients with anatomically complex degenerative mitral regurgitation (DMR), but long-term outcomes are not well known.

**Aims:**

To investigate outcomes by specific anatomical criteria in patients with severe DMR and complex valve anatomy enrolled in the prospective MitraUlm registry.

**Methods:**

Clinical and echocardiographic 3-year outcomes of 304 patients with DMR and complex mitral valve anatomy, defined according to the CLASP IID registry criteria, were investigated. Outcomes were analysed separately for specific anatomical complexities.

**Results:**

33.5% of patients had ≥ independent significant jets, 12% multisegmental prolapse, 12.3% mitral valve orifice area < 4 cm^2^, 10.9% commissural lesions with wide/multiple jets, and 10.1% large flail. Mitral regurgitation (MR) reduction ≤ 2 was achieved in 93.8% of patients at discharge and in 85.9% at 3-year follow-up. Patients with multisegmental prolapse and commissural lesions with multiple/wide jets had the lowest MR reduction at discharge. Compared to patients treated with MitraClip Generation 1–3, patients treated with MitraClip Generation 4 had significantly better post-procedural MR reduction (MR ≤ 1 in 74.3% vs 50.7%, *P* < 0.001) and higher 3-year survival rates (80.2% vs 61.6%, Log Rank *P* = 0.002*)*. Postprocedural MR reduction to MR ≤ 1 was the strongest independent predictor of 3-year survival, whereas the presence of at least two anatomical complexities and elevated post-procedural left atrial pressure predicted 3-year all-cause mortality.

**Conclusions:**

In patients with anatomically complex DMR, advances in procedural techniques for M-TEER have allowed successful treatment with sustained MR reduction and improved long-term survival.

**Graphical abstract:**

**Figure:**
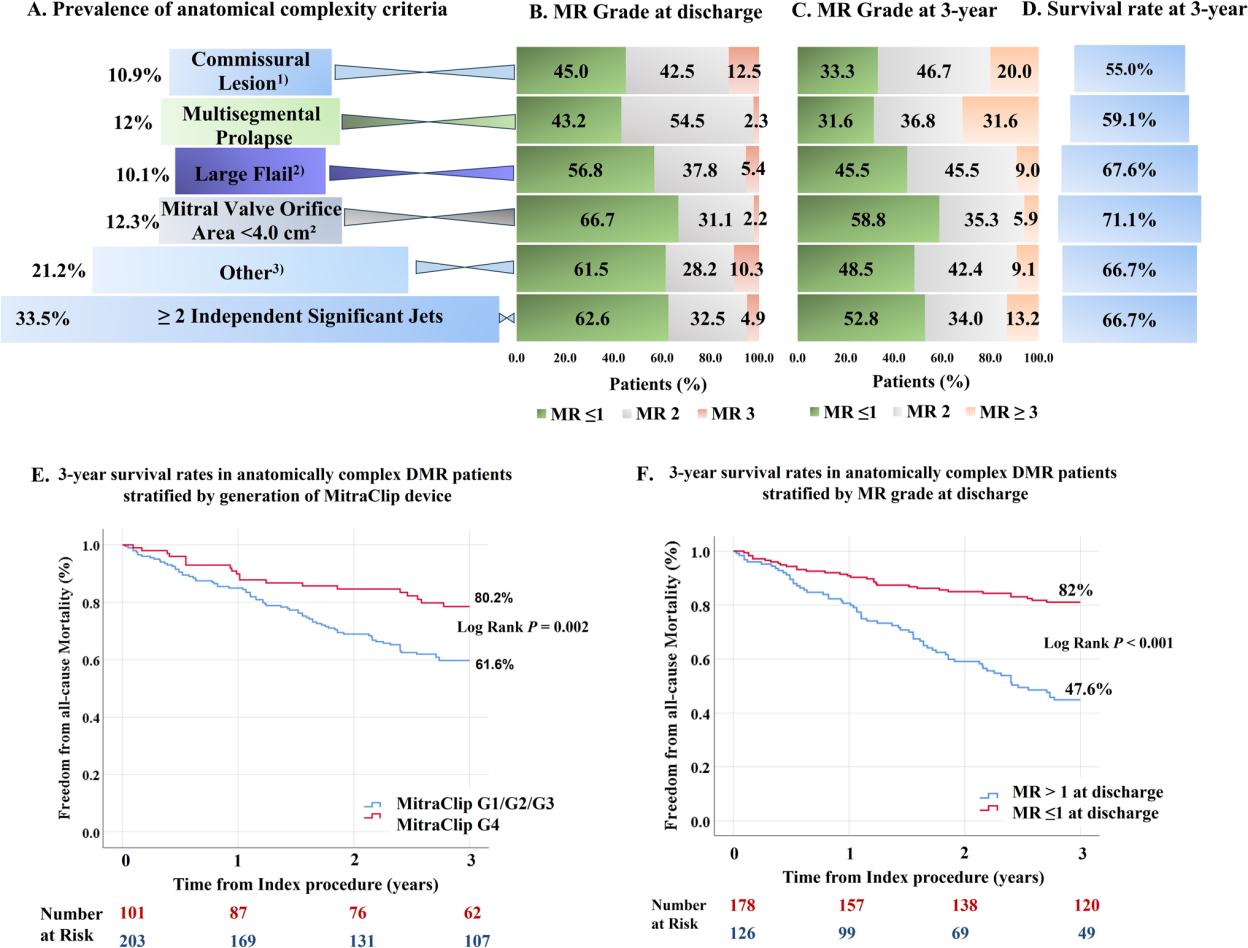
Central Illustration. 3-year Outcomes of M-TEER in anatomically complex DMR.** A.** Prevalence of anatomical complexity criteria. ^1)^ Patients with commissural lesions and wide/multiple jets; ^2)^ Large Flail is defined as flail width >15 mm and/or flail gap >10 mm. ^3)^ Other includes the following anatomical complexities: significant perforation or missing leaflet tissue in the grasping area, moderate to severe calcification in the grasping area and leaflet mobility length <8 mm;** B.** Mitral regurgitation reduction by specific anatomical complexities at discharge;** C.** Mitral regurgitation grade by specific anatomical complexities at 3-year follow-up;** D.** Survival rate by specific anatomical complexities at 3-year;** E.** and** F.** Kaplan Meier estimates for freedom from all-cause mortality stratified by MitraClip Generation (**E.**) and MR grade at discharge (**F.**);* MR* mitral regurgitation

## Introduction

Advances in procedural techniques for mitral valve (MV) transcatheter edge-to-edge repair (M-TEER) allow successful treatment of a growing population with degenerative mitral regurgitation (DMR) and complex anatomy, initially considered unsuitable for the procedure. The CLASP-IID registry recently reported sustained mitral regurgitation (MR) reduction and favorable survival at 1-year in 98 patients with DMR and heterogeneous anatomical complexities treated with M-TEER [1]. Despite promising short-term results, long-term outcomes in these patients are not well known and comparative analyses of outcomes by specific complexity criteria have not been published. The main purpose of the following study was to assess 3-year echocardiographic and clinical outcomes by specific anatomical complexity in patients with anatomically complex DMR enrolled in the prospective real-world MitraUlm registry over the last decade.

## Methods

### Study population

All patients had moderate to severe DMR, were deemed to be at prohibitive surgical risk by the local heart team and underwent M-TEER with the MitraClip device at our institution between January 2010 and June 2021. Complex valve anatomy was defined retrospectively according to the CLASP IID registry criteria, based on the anatomical recommendations in the MitraClip instructions for use [1]. Anatomical complexities included at least one of the following features: ≥ 2 independent jets, mitral valve orifice area < 4 cm^2^, multisegmental prolapse, commissural lesion with wide/multiple jets, flail width > 15 mm and/or flail gap > 10 mm, severe calcification in the grasping area, cleft in the grasping area, leaflet mobility length < 8 mm. There were no exclusion criteria for inclusion in our prospective registry. All patients gave informed consent to participate in the registry. The study protocol was approved by the ethics committee of the University of Ulm (435/16) and conforms to the Declaration of Helsinki.

### Study outcomes and echocardiographic evaluation

Procedural and acute outcomes were assessed according to the recommendations of the Mitral Valve Academic Research Consortium (MVARC). Long-term outcome data were evaluated at 3-year follow-up and included functional clinical assessment based on New York Heart Association (NYHA) classification, all-cause death, reintervention, rehospitalization and major adverse events, which included cardiovascular death, stroke, myocardial infarction and severe bleeding. Follow-up was conducted by routine clinical visit or telephone contact after device implantation.

Anatomical complexities were evaluated retrospectively using the screening transesophageal echocardiography (TEE) by a team of experienced cardiologists trained in interventional imaging and certified in TEE (EACVI certification). Follow-up echocardiography including MR grade was performed by transthoracic echocardiography (TTE). Clinical and echocardiographic outcomes were separately analyzed by specific anatomical characteristics.

### Statistics

Categorical variables were expressed as absolute numbers and percentages and compared by X^2^ or Fisher’s test. Normality of distribution of continuous variables was analyzed using Kolmogorov–Smirnov and Shapiro–Wilk tests. Continuous variables were presented as mean with standard deviation and compared using Student’s t-test or Mann–Whitney–Wilcoxon test. Optimal cut-off values for mean left atrial pressure with an area under the curve greater than 0.7 associated with an increased mortality risk were generated from the receiver operating characteristic analysis using the Youden threshold. Cumulative 3-year all-cause mortality rates were estimated using the Kaplan–Meier method, and the differences between subgroups were analyzed using the Log-rank test. Missing values were not imputed. Multivariable Cox regression analysis using stepwise forward selection was performed to assess the influence of relevant baseline and procedural variables on 3-year mortality. The algorithm was applied to all potentially influential parameters (P < 0.10) from the univariate logistic regression analysis. Collinearity between parameters was analyzed using variance inflation factors. All tests were two-tailed, and a P-value < 0.05. was considered statistically significant. SPSS statistical package version 20.0 (SPSS Inc., Chicago, IL, USA) 9.3 (Cary, NC, USA) was used for calculations.

## Results

### Baseline characteristics

**Fig. 1:**
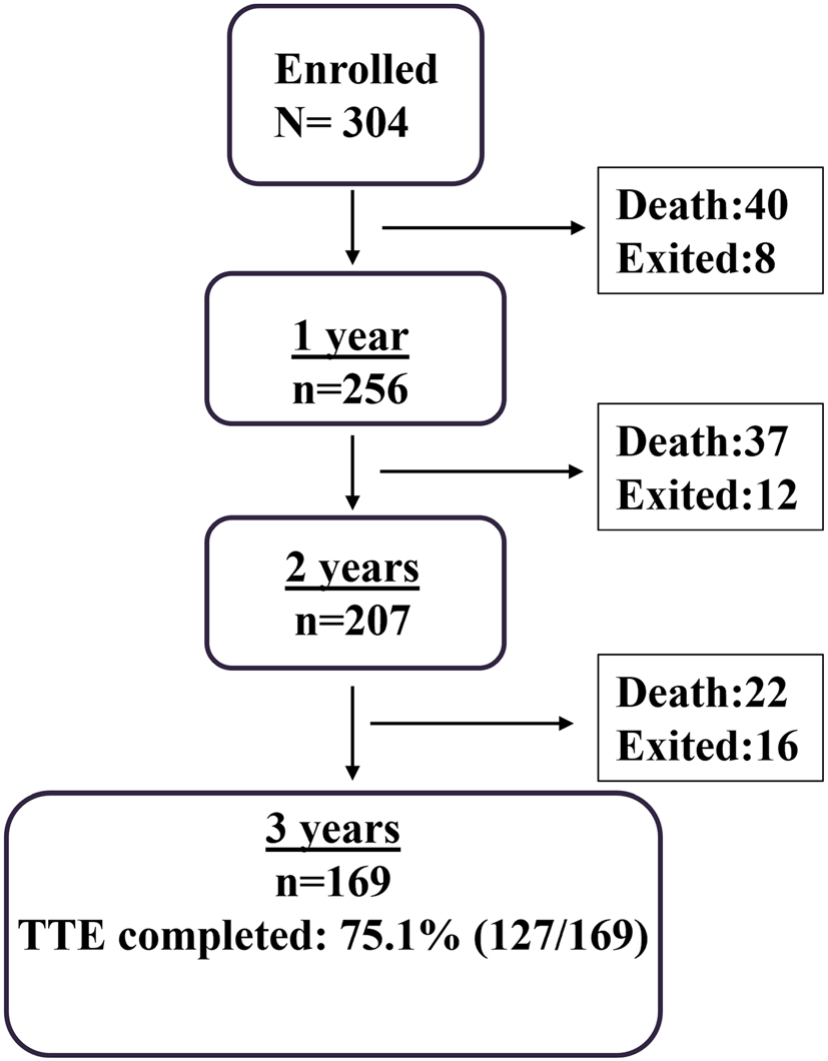
Flowchart of patients with anatomically complex DMR. Illustration of enrollment of patients with anatomically complex DMR in the MitraUlm Registry and follow-up at 1, 2 and 3 years; *DMR* degenerative mitral regurgitation, *TTE* transthoracic echocardiography

Of a total of 304 patients with DMR and complex valve anatomy enrolled in the prospective registry, 36 patients (11.8%) were lost to follow-up. There were no significant differences in baseline characteristics between patients lost to follow-up and those not lost to follow-up (Supplemental Table 2). Of the 169 patients eligible for 3-year follow-up, TTE was complete for 127 (75.1%, Fig. 1). The median follow-up duration was 1214 days. Patients were elderly (mean age 80 ± 7), predominantly male (61.5%) and 69.7% were in NYHA class III/IV. There was a high burden of comorbidities including coronary artery disease (48.4%), atrial fibrillation (60.2%), chronic kidney disease (50.3%) and diabetes mellitus (28%), as shown in Table 1.

**Table 1:** Baseline characteristics

Demographics	
Age (years)	80 ± 7
Male (%)	187 (61.5)
Euro score II	6.3 ± 3.2
NYHA cla**ss** III (%)	169 (55.6)
NYHA class IV (%)	43 (14.1)
Comorbidities/medical history	
Prior heart failure hospitalization	115 (37.8)
PCI (%)	107 (35.2)
CABG (%)	39 (12.8)
MV surgery (%)	5 (1.6)
Previous MI (%)	66 (21.7)
CAD (%)	147 (48.4)
DCM (%)	35 (11.7)
Hypertension (%)	246 (80.9)
Diabetes (%)	85 (28)
Atrial fibrillation (%)	183 (60.2)
Peripheral artery disease (%)	19 (6.3)
COPD (%)	38 (12.5)
Chronic renal failure (GFR < 60 ml/min, %)	153 (50.3)
Previous cancer (%)	57 (18.8)
Pacemaker/ICD (%)	47 (15.5)
Laboratory	
GFR (ml/min/1.73m^2^)	48 ± 19
NT-pro-BNP (pg/ml)	6646 ± 6590
Echocardiography	
DMR etiology	304 (100)
LVEF (%)	57 ± 8
LVEDD (mm)	57 ± 10
LVESD (mm)	38 ± 10
LA (mm)	56 ± 10
EROA (cm^2^)	0.46 ± 0.1
Mitral valve area (cm^2^)	5.4 ± 0.8
Mean transmitral gradient (mmHg)	2.5 ± 0.7
MR 3 +	88 (28.9)
MR4 +	216 (71.1)
TR ≥ moderate-severe	46 (15.1)
Catheterization	
Systolic PA pressure (mmHg)	50 ± 11
Mean LA pressure (mmHg)	23 ± 6
V wave LA pressure (mmHg)	39 ± 12

*CABG* coronary artery bypass grafting, *CAD* Coronary artery disease, *COPD* chronic obstructive pulmonary disease, *DCM* dilatative cardiomyopathy, *DMR* degenerative mitral regurgitation, *EROA* effective regurgitant orifice area, *GFR* glomerular filtration rate, *ICD* implantable cardiac defibrillator, *LA* left atrium, *LVEF* left ventricular ejection fraction, *LVEDD* left ventricular end-diastolic diameter, *LVESD* left ventricular end-systolic diameter, *MI* myocardial infarction, *MV* mitral valve, *NYHA* new york heart association, *PA* pulmonary artery, *PCI* percutaneous coronary artery intervention, *TR* tricuspid regurgitation

### Echocardiographic and invasive hemodynamic assessment

Echocardiographic assessment at baseline revealed a mean left ventricular ejection fraction of 57 ± 8%, the mean mitral valve area was 5.4 cm^2^ and the mean mitral gradient was 2.5 mmHg (Table 1). The most common anatomical complexities were the presence of ≥ 2 independent jets (33.5%), mitral valve orifice area < 4 cm^2^ (12.3%) followed by multisegmental prolapse (12%) and commissural lesions with wide/multiple jets (10.9%), as shown in Central Illustration A and Supplemental Table 1. 21.2% of the patients had very complex valve anatomies including cleft, severe calcification in the grasping zone, short leaflet length < 8 mm or large tissue defect in the leaflet. Complexity criteria did not change significantly over time except for the prevalence of cleft, which increased significantly from 2.8% in patients treated with the first, second and third Generation MitraClip to 10.7% in patients treated with the fourth-Generation MitraClip, Supplemental Table 1. 241 patients (79.3%) had one anatomical complexity, whereas 20.7% of patients had two anatomical complexities. Invasive hemodynamic measurements showed elevated systolic pulmonary artery pressures (50 ± 11 mmHg) and mean left atrial pressures (23 ± 6 mmHg).

### Periprocedural outcomes in anatomically complex DMR

Procedural and 30-day outcomes are shown in Table 2. Successful device implantation was achieved in 92.4% of patients. The mean number of implanted clips per patient was 1.6, with a mean device time of 84 minutes and a mean fluouroscopy time of 25 minutes. MitraClip Generation 1, 2 and 3 were implanted in 66.8 % of patients, whereas 33.2% of patients received MitraClip Generation 4. A total of 75.2 % of patients treated with MitraClip Generation 4 received wide clips, either NTW or XTW alone or in combination with the other 2 clips (NT or XT). The 30-day MACCE rate was 8.9 % and the 30-day all-cause mortality rate was 4.3%.

**Table 2:** Procedural and 30 days outcomes

Time after procedure (days)	6 ± 5.1
ICU length (days)	0.9 ± 3.5
Fluoroscopy time (min)	25 ± 10
Total device time (min)	84 ± 19
Number of clips implanted	1.6 ± 1
MVARC Device success	281 (92.4)
Postprocedural Mean LA pressure (mmHg)	18 ± 2.7
Postprocedural v wave LA pressure (mmHg)	27 ± 3.3
Implanted device	
MitraClip Generation 1/2/3	203 (66.8)
MitraClip Generation 4	101(33.2)
Periprocedural mortality	8 (2.6)
30 days rehospitalization	14 (4.6)
30 days all-cause mortality	13(4.3)
30 days MACCE	27 (8.9)

*ICU* intermediate care unit, *LA* left atrium, *MACC* major adverse cardiac and cerebrovascular events, *MR* mitral regurgitation, *MVAR* mitral valve academic research consortium

### Echocardiographic outcomes

**Fig. 2:**
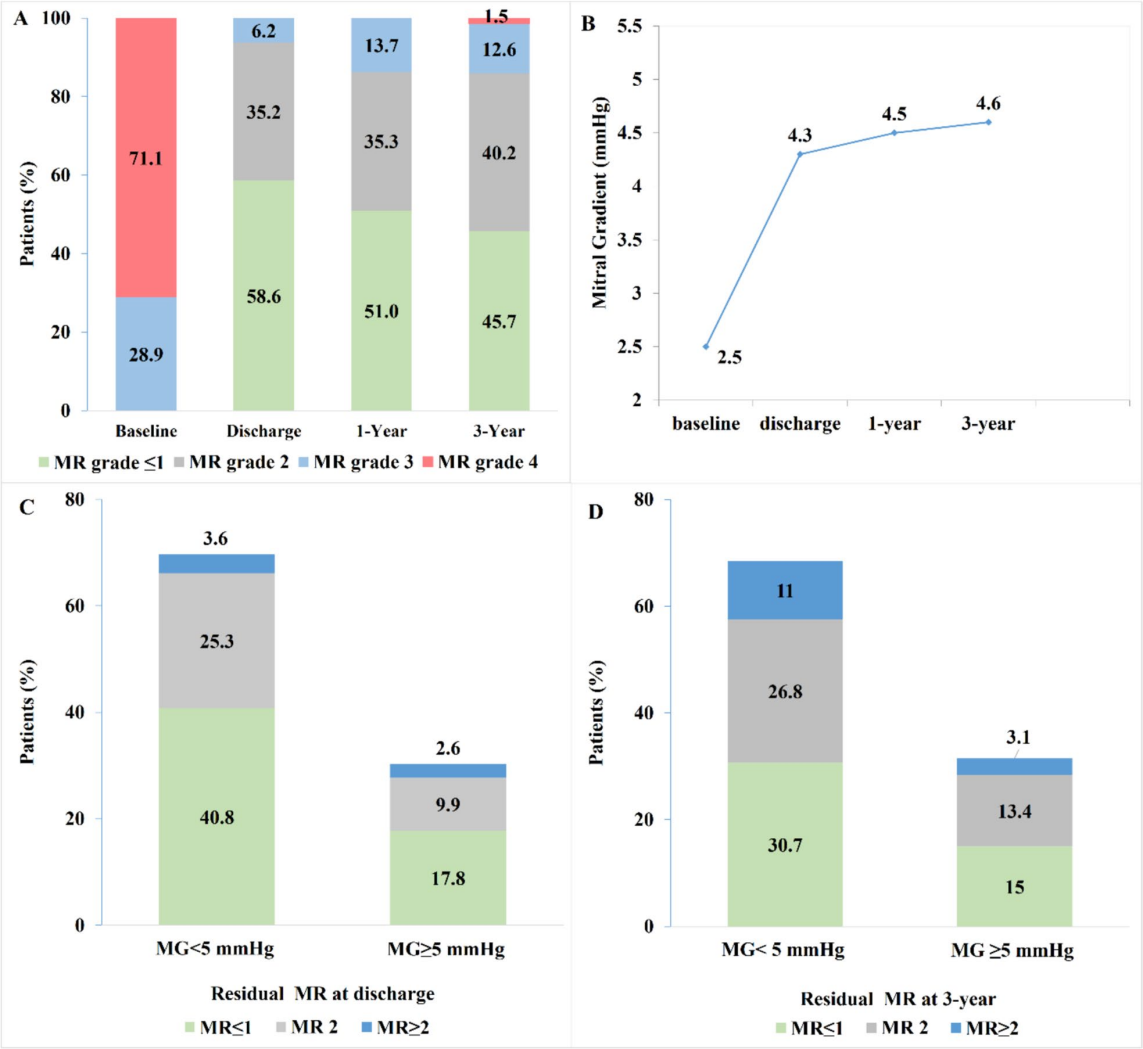
MR Grade and Transmitral Valve Gradient in Anatomically Complex DMR. Figure shows MR grade and transmitral gradient at discharge and at follow-up. **A**. Percentual distribution of MR grade at discharge, 1-year and 3-year follow-up; **B** Transmitral gradient at discharge and at 3-year follow-up; **C** and **D** Mitral regurgitation and transmitral valve gradient at discharge and at 3-year follow-up; Proportions represent patients within the MR severity and gradient groups of: MG < 5 mmHg with MR grade ≤ 1, MR grade 2, MR grade ≥ 2 and MG ≥ 5 mmHg with MR grade ≤ 1, MR grade 2, MR grade ≥ 2. *DMR* degenerative mitral regurgitation, *MG* mitral gradient, *MR* mitral regurgitation

At discharge, 93.8% of patients presented with MR ≤ 2 and 58.6% had MR ≤ 1. At 3 years, these proportions were 85.9% and 45.7% (Fig. 2A). Patients treated with the fourth-generation MitraClip system had a significantly higher proportion of MR ≤ 1 compared to patients treated with the first-, second- and third-generation MitraClip devices both at discharge (74.3% vs 50.7%, *P* < 0.001) and at 3-year follow-up (56.5% vs 39.5%, *P* = 0.064). Patients with multisegmental prolapse and commissural lesions with wide/multiple jets had the lowest MR reduction at discharge (MR ≤ 1 in 43.2% and 45%, respectively) and at 3-year follow-up (31.6% and 33.3%, respectively), whereas MR reduction to grade ≤ 1 at discharge for all other MV pathologies ranged from 56.8% in patients with large flail to 66.7% in patients with MV orifice area < 4 cm^2^ (Central Illustration B, C). Patients with multisegmental prolapse and commissural lesions treated with the fourth-generation MitraClip had better MR reduction compared to patients treated with the first-, second- and third-generation MitraClip devices (MR ≤ 1 in 63.6% vs 37.9%, *P* = 0.135 for commissural lesions and 58.8% vs 40.7%, *P* = 0.280 for multisegmental prolapse).

**Fig. 3:**
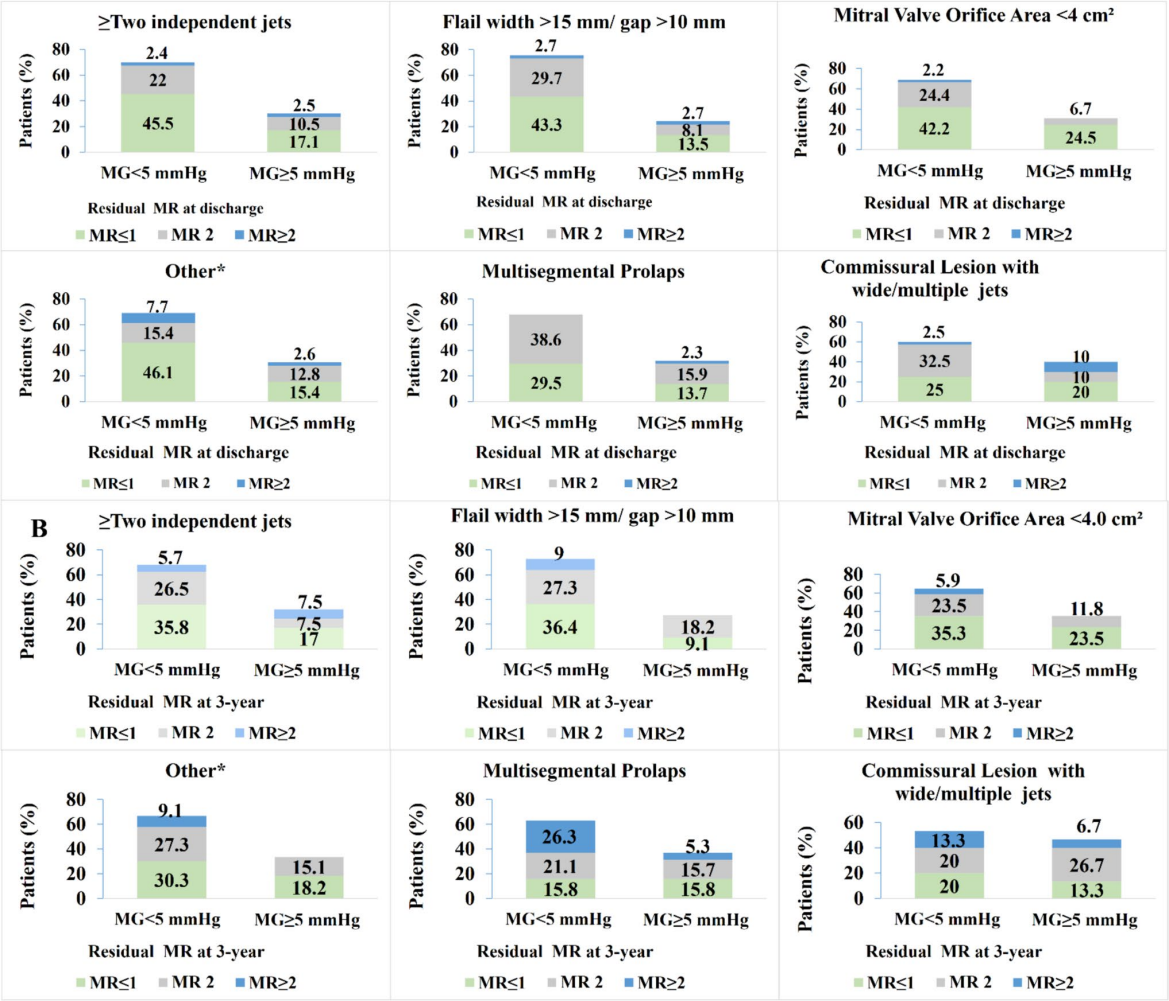
MR Grade and Transmitral Valve Gradient by specific anatomical complexities. Mitral regurgitation and transmitral valve gradient by anatomical complexity at discharge. Proportions represent patients within the MR severity and gradient groups of: MG < 5 mmHg with MR grade ≤ 1, MR grade 2, MR grade ≥ 2 and MG ≥ 5 mmHg with MR grade ≤ 1, MR grade 2, MR grade ≥ 2. * Other includes the following anatomical complexities: significant perforation or missing leaflet tissue in the grasping area, moderate to severe calcification in the grasping area and leaflet mobility length < 8 mm. *MG* mitral gradient, *MR* mitral regurgitation

The mean mitral gradient increased from 2.5 mmHg at baseline to 4.3 mmHg at discharge, with no significant increase at 3-year follow-up (Fig. 2B). Patients with commissural lesions with wide/multiple jets had the highest mitral gradients at discharge (40% of patients had gradients ≥ 5 mmHg) and at 3-year follow-up, whereas only 31.2% of patients with MV orifice area < 4 cm^2^ at baseline had mitral gradient ≥ 5 mmHg at discharge, as shown in Fig. 3A, B. MR ≤ 2 with mean gradient < 5 mmHg was achieved in 66.1% of all patients at discharge and in 57.5% of patients at 3-year follow-up. MR ≤ 1 and mean gradient < 5 mmHg was achieved 40.8% of all patients at discharge and 30.7% at 3-year follow-up, as shown in Fig. 2C,D.

### Functional and clinical 3-year outcomes

**Fig. 4:**
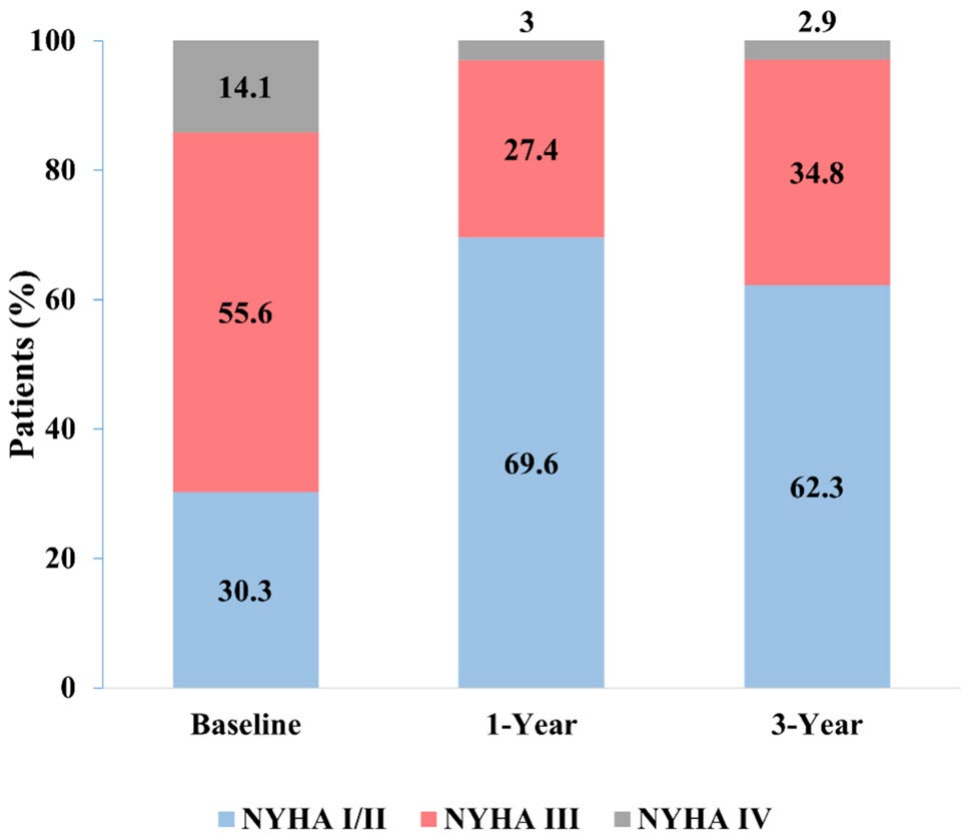
Functional Outcomes in anatomically complex DMR. NYHA functional class at baseline, 1-year and 3-year follow-up. *NYHA* new-york heart association

A significant improvement in NYHA functional class from baseline to 3-year follow-up was observed for all patients with anatomically complex DMR (Fig. 4). The proportion of patients with NYHA functional class I/II increased from 30.3% at baseline to 69.6% at 1-year and remained stable (62.3%) at 3 years.

Major adverse events, all-cause mortality, rehospitalization and reintervention rates at 3 years in patients with anatomically complex DMR are provided in Table 3. Major adverse events were reported in 37.5% of patients and hospitalization for heart failure in 28% of patients. Cardiovascular death was the most frequent major adverse event, followed by severe bleeding. The 3-year Kaplan–Meier estimate of freedom from all-cause mortality in the overall cohort was 67.4%. Survival rates by specific anatomical characteristics are depicted in the Central Illustration D. In the entire cohort, patients with multisegmental prolapse and commissural lesions had the lowest survival rates at 3–year: 59.1% and 55% respectively. Mortality rates in patients with other anatomical complexities ranged between 28.9% in patients with MV orifice area < 4 cm^2^ and 33.3% in those with ≥ 2 independent jets. Patients with very challenging valve anatomy such as cleft or severe calcification in the grasping area had a 3-year mortality rate of 33.3%. Patients treated with the fourth-generation MitraClip system had a significantly higher 3-year survival rate compared to patients treated with the first-, second- and third-generation MitraClip devices (80.2% vs 61.6% Log Rank *P* = 0.002, Central Illustration E). Significant improvements in 3-year survival with the fourth MitraClip Generation were also observed in patients with commissural lesions with wide/multiple jets (72.7% vs 51.7%, *P* = 0.230) and multisegmental prolapse (70.6% vs 51.9%, *P* = 0.218). Patients with MR ≤ 1 at discharge had significantly higher survival rates at 3 years compared to patients with MR > 1 at discharge (82% vs 47.6%, Log Rank *P* < 0.001, Central Illustration F). 9.5% of patients with anatomically complex DMR required reintervention after a median time interval of 5.5 months.

**Table 3:** Major adverse events at 3-year follow-up

Major adverse events	114(37.5)
Cardiovascular death	81(26.6)
Stroke	14(4.6)
Myocardial infarction	13(4.3)
Severe bleeding	35(11.5)
All-cause death	99(32.6)
Heart failure hospitalization	85(28)
Mitral valve reintervention *	29(9.5)

Kaplan-Mayer estimate (%) was used for 3-year outcomes. Mitral Valve reintervention * either surgical or percutaneous

### Predictors of all-cause mortality and reintervention

Clinical, echocardiographic and hemodynamic characteristics related to all-cause mortality by univariate and multivariate analysis are detailed in Supplemental Table 3 and Table 4. The presence of at least two anatomical complexities, post-procedural left atrial pressure ≥ 20 mmHg and pulmonary hypertension at baseline are significantly predictive of 3-year all-cause mortality, whereas MR reduction to grade ≤ 1 was independently associated with increased 3-year survival (HR 5.55, 95% CI 2.38–12.5, *P* < 0.001). Supplemental Table 4 and Table 4 summarize the results of the univariate and multivariate analyses for the prediction of reintervention. The presence of more than two anatomical complexities, large flail and MR grade > 2 at discharge were the strongest independent predictors of reintervention after the index procedure.

**Table 4:** Multivariable predictors for all-cause mortality and reintervention

Predictors of 3-year all-cause death	HR (95% CI)	P-value
MR Grade ≤ I at discharge	0.18(0.08–0.42)	** < 0.001**
Presence of ≥ 2 anatomical complexities	2.61 (1.11–6.62)	**0.042**
Postprocedural Mean LA pressure ≥ 20 mmHg	4.14 (1.27–9.46)	**0.018**
Systolic PA pressure (mmHg)	1.04 (1.01–1.08)	**0.039**
GFR (ml/min)	0.97(0.95–0.99)	**0.02**
Postprocedural mitral gradient ≥ 5 mmHg	1.74 (0.73–4.11)	0.207
NYHA class IV at baseline	1.97 (0.69–5.62)	0.146
EuroScore	1.09 (0.95–1.23)	0.193
MitraClip Generation 4	1.36 (0.43–2.48)	0.537
Commisural lesion with wide/multiple jets	2.68(0.88–8.12)	0.185

Predictors of 3-year reintervention	HR (95% CI)	P-value
MR Grade > 2 at discharge	4.1 (2.21–10.28)	**0.012**
Presence of ≥ 2 anatomical complexities	3.23(1.26–8.51)	**0.041**
Flail Gap > 10 mm and/or Width > 15 mm	5.75 (1.21–9.13)	**0.028**
Postprocedural Mean LA pressure ≥ 20 mmHg	2.50 (0.42–14.81)	0.312

The table shows predictors for all-cause mortality and reintervention for the whole population of 304 patients with degenerative mitral regurgitation and anatomically complex anatomy

*GFR* glomerular filtration rate, *LA* left atrium, *MR* mitral regurgitation, *NYHA* new york heart association, *PA* pulmonary artery

## Discussion

Improvements in procedural techniques over the last decade have increasingly spread the indications of M-TEER for the treatment of complex anatomies in degenerative mitral regurgitation in high volume centers. The prospective multinational CLASP IID registry published 1-year outcome results in 98 patients with anatomically complex DMR treated with the Pascal M-TEER-system showing favorable survival, sustained significant MR reduction and improvement in functional status [1]. We report 3-year clinical and echocardiographic outcomes in a large cohort of 304 patients with anatomically complex DMR enrolled in the prospective real-world MitraUlm registry over the past decade and present predictors of long-term mortality in this population initially considered unsuitable for percutaneous edge-to-edge treatment. This study focused on clinical and echocardiographic outcomes according to specific anatomical complexities. The main findings of our study are as follows: (1) the majority of patients showed sustained MR reduction with low gradients at 3-year follow-up; (2) post-procedural MR reduction to grade ≤ 1 and 3-year survival rates improved significantly in patients treated with the fourth-generation MitraClip system compared to patients treated with the first-, second- and third-generation MitraClip devices.

The prevalence of specific anatomical complexities in our registry are similar to those in the STS/ACC TVT real-world registry and the prospective multinational CLASP IID registry [2, 3], allowing for a proper comparison of outcomes.

In this real-world large cohort with anatomically complex DMR, we report a significant postprocedural MR reduction to MR ≤ 2 in 93.8% of patients which was sustained in 85.9% of patients at 3-year follow-up. MR reduction to grade ≤ 2 was the acceptable procedural goal in the early stages of M-TEER and most large trials reported high rates of MR ≤ 2 after the procedure. However, MR reduction ≤ 1 was significantly less common in the historical trials and only recent studies have highlighted the prognostic importance of MR reduction to grade ≤ 1 [4]. However, the prognostic value of postprocedural MR reduction to MR ≤ 1 in patients with complex anatomy has not been previously reported. The proportion of patients with MR ≤ 1 at discharge in our registry improved significantly from 50.7% in patients treated with the first, second and third Generation MitraClip devices to 74.3% in patients treated with the fourth Generation MitraClip devices. Importantly, these postprocedural results in patients treated with the fourth Generation MitraClip devices were maintained at 3-year follow-up in 56.5% of patients. Our results suggest that the development of single-grasping technology in last-generation devices and changes in device steering significantly improved procedural success. This is reassuring considering that more than 20% of patients had at least two anatomical complexities at baseline and more than 20% of patients had very complex leaflet features, with an increasing prevalence of cleft and severe calcification in recently treated patients. These recent post-procedural results are comparable to those of the CLASP IID registry, which reported a reduction in MR to grade ≤ 1 in 69.5% of patients at discharge and 57.6% at 1-year. The MR reduction in the MitraUlm registry, although similar to the CLASP IID registry, is poorer and less sustained compared to the recently published EXPAND G4 trial, which reported MR reduction to grade ≤ 1 at 1-year in 88% of patients with DMR, however, only one third of patients included in the EXPAND G4 trial had complex leaflet anatomy. Our results underline the importance of real-world data, as unselected, all-comer patients tend to have inferior outcomes [5]. Nevertheless, it is important to note that more recently patients were treated earlier in the course of disease and tend to have a better hemodynamic status at baseline with less severe pulmonary hypertension and cardiac dilatation which are known to affect the risk of MR recurrence. The prognostic importance of adequate MR reduction in this cohort with anatomically complex DMR was highlighted by multivariate analysis which identified MR grade ≤ 1 at discharge as the main independent predictor of 3-year survival. The improvement in procedural results resulted in better functional status.

To our knowledge, this is the first study to analyze long-term echocardiographic and clinical outcomes by specific anatomical complexity in patients with anatomically complex DMR treated with M-TEER. Patients with multisegmental prolapse, including those with Barlow syndrome, and patients with commissural lesions had the poorest MR reduction at discharge and the highest 3-year mortality rates among all patients with anatomically complex DMR. It should be noted that patients with commissural lesions in this registry had multiple or wide jets and the interventionalist had to compromise between sufficient MR reduction and postprocedural stenosis, as patients with commissural lesions indeed had the highest postprocedural mitral gradients of all complex pathologies treated with M-TEER. To date, several strategies have been attempted to reduce residual MR in commissural lesions including implantation of the Amplatzer Vascular Plug II occluder between the commissure and the M-TEER device. These bailout strategies proved poor short-term outcomes and have been abandoned [6]. The technical improvement of newer M-TEER devices with independent grasping features, leaflet optimization and narrower dimensions supported by advanced imaging have improved significantly the MR-reduction in patients with commissural MR treated in this real-world registry. Patients with multisegmental prolapse, especially those with Barlow syndrome, still pose a significant procedural difficulty, as many patients present besides tissue redundancy other features such as flail, cleft-like lesions and calcification in the grasping area which make qualitative grasping very difficult. Application of a positive end-expiratory pressure by mechanical ventilation, adenosine induced asystole, and an anchoring strategy have been used in some centers to allow the positioning of multiple clips with satisfactory grasping. The choice of device plays an important role in treating patients with prolapse and data from the EXPAND trial showed a preferential use of the XTR MitraClip over the NTR [7]. In our experience, the use of larger devices such as the XTR or Pascal P10 provides a better filling of the regurgitant orifice but may exert excessive leaflet tension in patients with poor leaflet quality, resulting in insufficient MR reduction in long-term follow-up. Interestingly, patients with MV orifice area < 4 cm^2^ and those with isolated very complex leaflet features including cleft or short leaflets showed satisfactory MR reduction at 3 years.

The present study reports 3-year clinical outcomes including mortality and major adverse events in an unselected real-world population with anatomically complex DMR. The 3-year Kaplan Meier estimate of freedom from all-cause mortality in the overall cohort was 67.4%. Patients treated with the fourth-generation MitraClip device had significantly higher 3-year survival rates compared to patients treated with the first-, second- and third-generation MitraClip devices (80.2% vs 61.6%). There is limited data on outcomes by specific anatomical complexity in patients undergoing M-TEER [8]. Although patients with commissural lesions with wide/multiple jets and multisegmental prolapse had the lowest survival rates among all patients (55% and 59.1%, respectively), patients treated with the latest generation MitraClip devices benefit from improved 3-year survival rates from 51.7 to 72.7% in patients with commissural lesions and from 51.9 to 70.6% in patients with multisegmental prolapse. These results reflect the technical improvement of the devices, the increasing expertise of interventionalists and imagers, but also the strategic shift toward treating patients at earlier stages of the disease.

In addition to post-procedural MR grade, multivariate analysis identified the presence of at least two anatomical complexities and elevated post-procedural mean left atrial pressures as independent predictors of 3-year all-cause mortality in patients with complex anatomy. The presence of at least two anatomic complexities had a direct impact on 3-year survival in this cohort, suggesting that these patients should be treated in centers with sufficient expertise. Elevated post-procedural left atrial pressure after M-TEER reflects the hemodynamic impact of both increased mitral gradients and relevant residual MR and has been associated with poor outcomes in previous studies [9] and in this population with anatomically complex DMR. Newer M-TEER devices have been designed to facilitate left atrial pressure monitoring during the procedure, which can significantly impact long-term outcomes. It is important to highlight the relatively low prevalence of elevated postprocedural mitral gradient ≥ 5 mmHg at 3 years in this cohort with anatomically complex DMR (29.9%), especially in patients with MV orifice area < 4 cm^2^ at baseline (35.3%), which is comparable to patients without complex leaflet anatomies.

Reintervention after index M-TEER is a challenging procedure associated with increased mortality [10]. Patients with anatomically complex DMR had a relatively low reintervention rate of 9.5% at 3 years. The presence of at least two anatomical complexities and large flail were independently associated with increased reintervention and may serve as parameters to identify patients who require careful strategic planning before the index procedure.

### Study strengths and limitations

This study reports long-term outcomes from a large real-world cohort with anatomically complex DMR and is the first study to report time-to-event outcomes by specific anatomical complexity. The development of single grasping technology of the latest generation MitraClip devices improved significantly the procedural success in patients with anatomically complex DMR. Therefore, the G1-G3 generation MitraClip data may not necessarily reflect today’s outcome with the most recent devices. Also, it is difficult to discriminate effects of improved technology from a longer learning curve, as the growing expertise of both interventionalists and imagers may have contributed significantly to improved outcomes. Considering the non-randomized nature of this all-comers registry, echocardiographic and follow-up data including soft endpoints like bleeding or myocardial infarction are site-reported, without adjudication by an independent event committee. Due to the large referral areas, sample size was limited during follow-up. Core lab TOE and TTE analysis was not available, and reports of MR reduction might be susceptible to observer bias. Finally, this is a single-centre study and external validation of our findings by larger prospective trials is needed.

## Conclusions

The results of this real-world registry provide an understanding of the evolution of M-TEER in patients with anatomically complex DMR over a decade and highlight the echocardiographic and outcome particularities of specific anatomical pathologies. Advances in M-TEER device technology and intraprocedural imaging have significantly improved echocardiographic and clinical outcomes over the past decade. Postprocedural results, particularly MR reduction to grade ≤ 1 improved significantly in patients treated with last-generation devices, leading to better long-term survival even in patients with multisegmental prolapse and commissural lesions with wide/multiple jets who presented low MR reduction and poor survival in the early M-TEER era. Postprocedural MR reduction to grade ≤ 1 was the strongest predictor of long-term survival. Interventionalists should therefore be diligent in achieving maximal MR reduction, even in patients with very challenging anatomy. The presence of at least two anatomical complexities was an independent predictor of long-term mortality suggesting that M-TEER in these patients should be performed in centres of expertise.

## Data Availability

The datasets generated and analyzed during the current study are available from the corresponding author on reasonable request.

## Abbreviations

DMR: Degenerative mitral regurgitation
MACCE: Major adverse cardiac and cerebrovascular events
MR: Mitral regurgitation
MV: Mitral valve
MVARC: Mitral valve academic research consortium
M-TEER: Mitral valve transcatheter edge-to-edge repair
NYHA: New york heart association
TEE: Transesophageal echocardiography
TTE: Transthoracic echocardiography
